# Therapy for HBV Infection in Hemodialysis Patients: Is it Possible?

**DOI:** 10.5812/hepatmon.834

**Published:** 2012-03-28

**Authors:** Behzad Einollahi

**Affiliations:** 1 Nephrology and Urology Research Center, Baqiyatallah University of Medical Sciences, Tehran, IR Iran

**Keywords:** Therapy, Patients, Infection

## 1. Introduction

Despite the use of preventive measures, particularly hepatitis B virus (HBV) vaccine, and recent advances in antiviral therapy, chronic HBV infection has still remained a public health concern among infected dialysis patients because it results in an increased risk of morbidity and mortality [1]. However, Alavian et al. have shown that the prevalence of HBV infection among hemodialysis (HD) patients in Iran declined to 2.4 - 2.6% in 2005 [2][3]. Furthermore, the clinical course and natural history of HBV infection are different in dialysis and nondialysis patients, and it may lead to a unique clinical problem in dialysis subjects. In addition, limited data are available on the optimal therapy of chronic HBV infection in dialysis patients. Therefore, in this article we discuss whether antiviral therapy is possible in infected dialysis patients with HBV.

## 2. Goals of Therapy

Clearance of HBsAg with anti-HBs seroconversion is an important aim of treatment for all HBV antiviral therapies. However, this goal can only be reached in a small proportion of immunocompetent patients, and reaching this goal is likely to be even less frequent in immunocompromised patients such as HD individuals [4]. Parameters used to assess treatment response include normalization of serum alanine aminotransferase (ALT; biochemical response), decrease in serum HBV DNA level below the lower limit of detection of sensitive polymerase chain reaction (PCR) assays, loss of HBeAg with appearance of anti-HBe (HBeAg seroconversion) in patients who are HBeAg positive at baseline, and improvement of liver histology (decline in necroinflammatory activity and no enhance in fibrosis; [5]. Moreover, the ultimate goal for treatment of chronic HBV is to prevent the development of irreversible complications such as cirrhosis, liver failure, and hepatic cancer. All dialysis patients using antiviral therapy against HBV infection should be monitored for response to therapy and for side effects of drugs. The serum ALT levels of dialysis patients should also be regularly monitored; unfortunately, the biochemical response to therapy is difficult to identify due to its depressed level in these patients [6]. Although a decrease in serum HBV DNA viral load during treatment appears to be the most important measure of response to therapy and improved long-term patient outcomes [7], HBV DNA levels are likely to be lower range in dialysis patients with HBV infection than in infected patients with normal renal function [8]. According to AASLD guidelines, all patients who are receiving therapy for HBV should have a liver panel measured every 3 months and HBV DNA levels quantified every 3 to 6 months [9]. In addition, patients should also be monitored every 3 to 4 months by serum alpha-fetoprotein levels and at least annually by liver ultrasound. Monitoring of HD patients with HBV infection during treatment is crucial for assessing drug safety, patient compliance, and treatment responses. Early monitoring of HBV DNA is of particular value to detect primary treatment failure and predict outcomes of sustained therapy (e.g., improved liver histology, prevention or reduced likelihood of disease progression, and occurrence of drug resistance). Suggestions for HBV DNA monitoring in patients on antiviral therapy are summarized in Table 1 [10].

**Table 1:** HBV DNA Monitoring During Antiviral Therapy

**Treatment duration, y**	**HBV DNA**	**Interpretation**
1	≥ 10 IU/mL or ≥ 50 copies/mL reduction from baseline	Virologic response
1	< 10 IU/mL or < 50 copies/mL reduction from baseline	Primary treatment failure
2	Undetectable by PCR	Complete virologic response
2	≥ 60 to < 2000 IU/mL (or ≥ 300 to < 10,000 copies/mL)	Partial virologic response
2	> 2000 IU/mL (or > 10,000 copies/mL)	Inadequate virologic response

## 3. Indications for Antiviral Therapy

Deciding which dialysis patients with HVB infection should be treated is a controversial issue. In the case of patients with normal renal function, antiviral therapy for chronic HBV infection is indicated in those with active virus replication (HBeAg positive and/or detectable serum HBV DNA) and active liver disease (raised ALT levels; [11][12]. However, a scarcity of data exists concerning the optimal therapy of chronic HBV infection in dialysis patients. It is obvious that these patients present a different clinical and biochemical picture in chronic HBV infection [13]. Dialysis individuals with HBV infection are less likely to have a symptomatic acute illness and are more likely to develop chronic carrier status [14]. In inactive carriers (i.e., dialysis patients with HBsAg and undetectable HBV DNA), there is no indication to start antiviral therapy, although monitoring for complications such as hepatocellular carcinoma should be undertaken [4]. On the other hand, serum ALT levels tend to remain within the normal range [15], and HBV DNA levels are likely to be lower in dialysis patients with HBV infection than in infected patients with normal renal function [8]. Therefore, in patients who are HBsAg-positive and have viral replication, a liver biopsy should be conducted, even in the presence of ALT within the normal range levels [4]. Finally, those with high HBV DNA values or evidence of active inflammation on biopsy are candidates for initiation of antiviral therapy [16].

## 4. Antiviral Agents

Seven drugs have received approval for the treatment of chronic HBV infection, including interferon-α, pegylated interferon-α, the nucleoside analogues lamivudine and telbivudine, and the nucleotide analogues adefovir, tenofovir, and entecavir. Entecavir, telbivudine, and tenofovir are the most potent, followed by lamivudine and then adefovir. Entecavir and tenofovir are associated with the lowest risk of drug resistance, followed by adefovir, telbivudine, and lamivudine, in that order [17].

## 5. Interferon-Alfa

Interferon-α, an antiproliferative and immunomodulatory agent, was the first available antiviral drug for chronic HBV infection. This agent is metabolized by renal tubules. In dialysis patients, it has been found that interferon- α's half-life is greatly enhanced, and prolonged treatment can lead to drug accumulation; hence, its adverse effects are magnified and include flu-like symptoms, nausea, diarrhea, fatigue, leucopenia, thrombocytopenia, thyroid dysfunction, alopecia, and depression [16]. Therefore, interferon-α is poorly tolerated by dialysis patients who have a frequent occurrence of side-effects, such as exacerbation of anemia, neutropenia, and protein malnutrition. Scarce data exist on treatment with interferon-α among infected dialysis patients with HBV. In one study, the adverse effects were so severe that withdrawal of the drug was required in more than 50% of patients [6]. Newer, pegylated interferon, an agent with a longer half-life, is no better tolerated in patients with renal failure. Finally, interferons are not recommended in dialysis patients with HBV infection [16].

## 6. Lamivudine

Lamivudine is the first nucleoside analogue antiviral approved worldwide for the treatment of chronic hepatitis B. Its major advantages include ease of administration (oral), potent antiviral activity, and favorable safety profile. Lamivudine has also been shown to be effective in the treatment of HBV-associated, acute glomerulonephritis [18]. Because lamivudine is primarily excreted via the kidneys and renal impairment could lead to a three or fourfold increase in its half-life, this drug targets the replication of the HBV genome and is well tolerated and safe with end-stage renal disease (ESRD) patients when the dose of drug is precisely adjusted for the degree of renal insufficiency. Good results were obtained in a series of 16 dialysis patients: 56% were able to eliminate HBV DNA and 36% were able to clear HbeAg [19]. The main disadvantages of lamivudine include risk for drug resistance and the need for a long duration of treatment. Despite its promising short-term efficacy, the widespread and long-term use of lamivudine monotherapy has led to a progressive increase in the numbers of patients with HBV resistance to lamivudine after prolonged treatment; for example, Fontaine et al. showed that lamivudine-resistant HBV develops in 39% of dialysis patients after a median of 16.5 months of treatment [20]. The prevalence of drug resistance enhanced with the duration of the therapy, and it was detectable in 14% of patients who were continuously treated for one year and in 69% of patients who were treated for 5 years [6]. Despite the risk of mutational resistance, lamivudine requires continued use for prolonged treatment [16]. On the other hand, the development of drug-resistant variants after prolonged therapy with lamivudine is a major limitation [12][20]. Nonetheless, there are still some problems with using lamivudine in dialysis patients. It is important to note that patients' treatment response could be assessed by serum ALT levels; however, serum transaminase levels tend to remain within the normal range or just slightly higher. In addition, the overall effects and outcomes of lamivudine therapy in dialysis patients remain unclear, and further studies are required for clarification [6]. The high occurrence of drug resistance may result in potentially life-threatening exacerbation of liver disease.

## 7. Adefovir Dipivoxil

Adefovir dipivoxil, a nucleotide analogue, is the second oral agent approved for the treatment of chronic HBV infection. It is usually added to therapy for lamivudine-resistant patients [21]. Adefovir is effective in patients with HBV infection who have normal renal function, although a very high relapse rate is seen if this drug is stopped; for example, relapse was observed in 92% of individuals with normal renal function in whom adefovir was discontinued after 48 weeks of the treatment [22]. Resistance is much less common than with lamivudine; 0% at one year and 29% at 5 years [23]. This makes adefovir an option for add-on therapy in patients who have developed lamivudine resistance [24]. Furthermore, adefovir is a nephrotoxic agent [6], and worsened renal function has been reported in 2.5 to 28.0% of cases after 1 to 2 years of therapy [6]. Limited data about adefovir administration exist in the chronic kidney disease (CKD) population [25][26]. Adefovir is eliminated via the kidneys; thus, a dose adjustment is required in CKD patients to prevent drug accumulation and its side effects [21]. In one study, adefovir was used in a series of 12 patients with CKD who had lamivudine-resistant HBV; a significant reduction was observed in HBV DNA viral loads after a median of 15 months of treatment [25]. Additionally, a case study described successful treatment of HBV infection in a dialysis-dependent liver-transplant recipient who had lamivudine-resistant infection and cirrhosis of the allograft [26].

## 8. Entecavir

Entecavir is a promising nucleoside analogue drug that has selective anti-HBV activity, especially against lamivudine-resistant HBV. Although no long-term data exist for lamivudine, the rate of entecavir resistance appears to be minimal, only about 1% after 3 years of monotherapy [27]. Entecavir is effective in viral suppression; however, the emergence of entecavir resistance with prolonged treatment could pose a dilemma [28][29]. In one study, entecavir treatment in subjects with normal renal function was associated with a significant decrease in HBV DNA viral load compared to lamivudine and adefovir [16]. Although the field has established little information on the therapeutic impact of entecavir in dialysis patients with chronic HBV infection, it is often recommended as a first-line oral therapy in patients with kidney disease [16]. Therefore, the optimal duration of treatment for these patients as well as the relationship between treatment and long-term outcomes such as cirrhosis and hepatocellular carcinoma remain unknown. Because entecavir is primarily eliminated by the kidneys, dose reduction is recommended for dialysis patients [30]. Moreover, the drug should be administered after hemodialysis, and continuous ambulatory peritoneal dialysis can remove approximately 0.3% of the dose over 7 days. It should be noted that entecavir has to be administered on an empty stomach (at least 2 hours after a meal or at least 2 hours before the next meal).

## 9. Tenofovir

The nucleotide analog tenofovir is recommended as a first-line oral antiviral in the treatment of chronic HBV patients with normal renal function [31]. It is the most potent agent against HBV infection within the first year of treatment [32]. Tenofovir can also be an effective alternative in patients with lamivudine-resistant HBV infection and has greater HBV DNA suppression, more normalized ALT levels, and a greater loss of HBsAg than adefovir [33]. However, nephrotoxicity and acute kidney injury have been reported in some patients treated with tenofovir [34][35][36][37][38]; therefore, the drug should be avoided in dialysis patients with residual renal function. A case report showed that one weekly dose of 300 mg of tenofovir was effective in a single HBV-infected dialysis patient [39]. This finding was replicated by the manufacturers of the drug in a study of 9 dialysis patients [40].

## 10. Telbivudine

Telbivudine is a synthetic thymidine nucleoside analogue that inhibits HBV DNA polymerase [41]. Telbivudine is a potent anti-HBV drug that provides effective and sustained viral suppression [42]. In clinical trials, telbivudine has been superior to lamivudine in HBV patients in terms of treatment outcomes and HBV DNA suppression after 1, 2, and 4 years of therapy [42][43]. When compared with lamivudine, telbivudine has greater HBV-DNA suppression (down to undetectable levels) at weeks 24 and 104, and HBeAg seroconversion rates are significantly greater with telbivudine than with lamivudine [44]. In addition, telbivudine-treated subjects have significantly less viral resistance than do lamivudine-treated individuals [9][42]. After 2 years of therapy, resistance to telbivudine emerged rarely in those who had a complete viral response at 6 months [9]. In a study on Chinese patients with chronic hepatitis B, telbivudine treatment for 52 weeks provided greater antiviral and clinical efficacy than lamivudine, with less resistance [45]. Telbivudine was also well tolerated in clinical trials for periods of up to 4 years with minor side effects [42]. Thus, telbivudine can be considered a valuable agent in the treatment of chronic HBV infection [42]. In a study that evaluated the effect of renal insufficiency on the pharmacokinetics of telbivudine, 36 cases received a single oral dose of telbivudine, adjusted on the basis of creatinine clearance. Telbivudine was well tolerated by all subjects. This study showed that the pharmacokinetics of telbivudine were dependent on renal function, especially for subjects with moderate to severe renal dysfunction or ESRD. These results indicated that although no adjustment of the telbivudine dose appears necessary for subjects with mild renal insufficiency, dose adjustment is warranted for those with moderate to severe renal impairment or ESRD in order to achieve optimal plasma exposure [46]. Telbivudine may be used for the treatment of chronic hepatitis B in patients who have impaired renal function. The drug is eliminated primarily through the kidneys; therefore, dose adjustment is recommended in patients with creatinine clearance of less than 50 mL/min, including dialysis patients. A hemodialysis session, routinely 3.5 to 4 hours, can reduce telbivudine from a patient's plasma by approximately 23% [46], and no additional dose modification is necessary during routine hemodialysis. Furthermore, telbivudine should be administered after hemodialysis sessions when given on hemodialysis days.

## 11. Conclusions

In summary, treatment of HBV infection is changing with time. Lamivudine is no longer regarded as the first-line treatment for chronic hepatitis B due to high rates of drug resistance. One cause of antiviral failure is the use of a less potent agent, such as adefovir [4], which seems to function better as an add-on therapy in patients who have developed lamivudine resistance. In addition, adefovir has not been well examined in dialysis patients. Although, tenofovir and entecavir are likely to be more effective and safer in dialysis patients, long-term empirical data are very limited on these drugs. It seems that entecavir can be administrated as a first-line oral therapy in dialysis patients, but progressive emergence of entecavir resistance with prolonged treatment could pose a problem. No data about telbivudine administration exist in dialysis patients. Finally, the best approach to managing these patient populations is still vaccination against HBV along with isolation of infected patients during hemodialysis sessions [1][47].
